# 678 Timing of Burn Care Access: A Single-Institutional Experience of Sociodemographic Determinants and Outcomes

**DOI:** 10.1093/jbcr/iraf019.307

**Published:** 2025-04-01

**Authors:** Elizabeth Boudiab, Artur Manasyan, Nicolas Malkoff, Brigette Cannata, Eloise Stanton, Maxwell Johnson, Haig Yenikomshian, Justin Gillenwater

**Affiliations:** Los Angeles General Medical Center Burn Unit; Keck School of Medicine University of Southern California; Keck School of Medicine University of Southern California; Keck School of Medicine University of Southern California; University of Southern California; Keck School of Medicine University of Southern California; Keck School of Medicine University of Southern California; Los Angeles General Medical Center

## Abstract

**Introduction:**

Ideal burn care requires prompt interventions such as wound and body temperature management, infection control, and fluid resuscitation. Delays in care can cause hypothermia and burn wound progression. In this study, we identify specific risk factors and outcomes associated with delayed admission to a regional burn center.

**Methods:**

Patients admitted with acute burn injury from 01/01/2019 to 12/31/2023 were included. Medical records were retrospectively reviewed for demographic data, timing of admission, burn injury characteristics, and clinical outcomes. Primary outcome variables included intensive care unit (ICU) stay, ventilator requirement, and mortality.

**Results:**

A total of 3,137 patients met inclusion criteria. 63% of patients were admitted within 24 hours, while 37% over 24 hours after injury. Male patients were likely to experience delayed admission (39.0 vs. 31.8%, p< 0.001). TBSA varied between the delayed and control cohorts (15.518.7% vs. 8.212.9%, p< 0.001). Patients who were single (p< 0.001) and lived alone (p=0.011) were more likely to experience a delay in burn unit admission. Homelessness (p< 0.001), substance abuse disorder (p< 0.001), and uninsured status (p< 0.001) were also associated with delayed admission. The delayed admission cohort underwent more surgeries (p=0.025), had a greater proportion of patients requiring mechanical ventilation (p< 0.001), and were more often admitted to the ICU (p< 0.001). In regression analysis when controlling for total body surface area (TBSA), delay in care was significantly associated with greater requirement for ICU stay (p< 0.001) stays and mechanical ventilation (p=0.021) but was not associated with increased mortality (p=0.232).

**Conclusions:**

Sociodemographic variables such as homelessness, lack of social support, and substance abuse are risk factors for delayed burn unit admission. Delayed admission of critically injured burn patients results in increased morbidity and hospital expenditures.

**Applicability of Research to Practice:**

Knowledge of these risk factors can inform future interventions to improve outcomes for vulnerable patients, promoting better recovery and long-term outcomes after burn injury. Future studies should utilize root-cause-analysis to examine the specific causes for delayed admission to optimize outcomes.

**Funding for the Study:**

N/A

